# Posterior maxillary segmental osteotomy for management of insufficient intermaxillary vertical space and intermolar width discrepancy: a case report

**DOI:** 10.1186/s40902-016-0074-0

**Published:** 2016-07-25

**Authors:** SeungWoo Baeg, SungWoon On, JeongKeun Lee, SeungIl Song

**Affiliations:** Department of Oral and Maxillofacial Surgery, Institute of Oral Health Science, Ajou University School of Medicine, 164, Wroldcup-ro, Yengto-gu, Suwon-si, Gyeonggi-do 16499 Republic of Korea

**Keywords:** Insufficient vertical space, Prosthetic rehabilitation, Orthognathic surgery, Posterior maxillary segmental osteotomy

## Abstract

**Backgrounds:**

Insufficient intermaxillary space is caused by non-restoration following tooth extraction in the past, and this involves eruption of the opposing teeth and changes of the arch structure. Such cases are difficult just by a simple prosthetic approach, and diversified treatment plans should be established. Among these, posterior maxillary segmental osteotomy (PMSO) is an efficient treatment option than extraction of opposing teeth as it surgically repositions multiple erupted teeth and alveolar bone. PMSO can preserve the natural teeth; therefore, it is being regarded as a treatment method which can improve insufficient intermaxillary space significantly.

**Case presentation:**

In this case report, the first patient received PMSO in order to place an implant in the mandibular edentulous space after decreased vertical dimension is restored, and the second patient received PMSO along with orthodontic treatment to obtain the intermaxillary space and balance the interarch molar width.

**Conclusion:**

PMSO is the treatment of choice when occlusion is compromised in the presence of decreased vertical dimension or arch length discrepancy.

## Background

If the extraction side is not restored in a proper time following tooth extraction, the opposing tooth is erupted, and shortening of vertical dimension for prosthetic treatment occurs [1]. In this case, securing of insufficient intermaxillary space for fixed or removable prosthesis can be an important point in establishing treatment plan [1, 2].

Severely erupted maxillary molar teeth after mandibular tooth extraction make reconstructing balanced intra-oral environment difficult. There are a few kinds of methods to improve the decreased intermolar space. When the extrusion of teeth is not too severe, it is possible to regain the space by coronoplasty procedure accompanying molar endodontics or periodontal crown lengthening with endodontic treatment of the maxillary teeth. To correct occlusion, orthodontic intrusion can be a treatment option using conventional orthodontic methods or mini-implants as a skeletal anchorage. When the extrusion is too severe, tooth extraction of the residual maxillary molar teeth is often proposed.

Maxillary molar segmental osteotomy can be used as a surgical procedure to significantly restore previously decreased vertical dimension following extraction of the mandibular molar tooth which is expected to have poor prognosis. And it is being suggested as a treatment method to enable prosthetic restoration using further mandibular molar implant-supported fixed or removable prosthesis [3–5].

This case presentation covers the following two cases. The first patient had severely extruded maxillary molars which have been left for a long time after extraction of the opposite teeth. The second patient had a scissor bite and the extrusion of the opposite side of maxillary molar teeth due to unilateral chewing habit. Both cases were successfully treated with PMSO.

## Case presentation

### Case 1

**Fig. 1:**
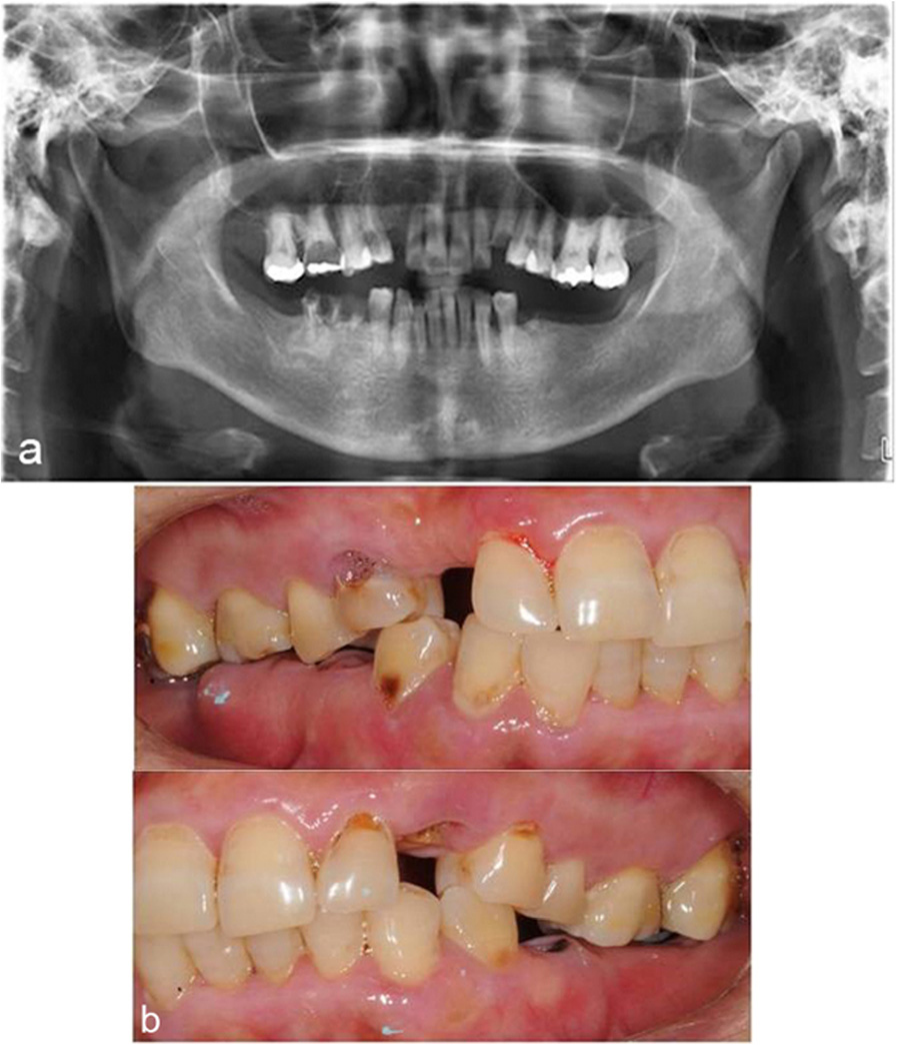
Preoperative panoramic view (**a**) and intra-oral photo of case 1 showing vertical dimension on the first molar 0 mm (**b**)

A 55-year-old female patient with serious bilateral maxillary molar eruption visited the hospital to secure space for mandibular molar prosthetics. For diagnosis, she received clinical and radiological examinations (Fig. 1).

She had serious bilateral maxillary molar eruption (bilateral vertical dimension on the first molar 0 mm), bilateral mandibular molar edentulous condition, lack of proper vertical dimension for prosthetic treatment, and multiple dental caries, and retained dental root were observed.

PMSO was planned to restore decreased vertical dimension due to serious eruption of the bilateral maxillary molar, and we decided to shift the molars in a posterior-superior direction by approximately 7 mm from the maxillary and mandibular right first premolar.

Incision was conducted in accordance with local analgesia under general anesthesia, and a buccal flap was formed. Horizontal osteotomy was performed from the bilateral first premolar to the first permanent molar, while vertical osteotomy was conducted on the mesial surface of the bilateral first premolar.

**Fig. 2:**
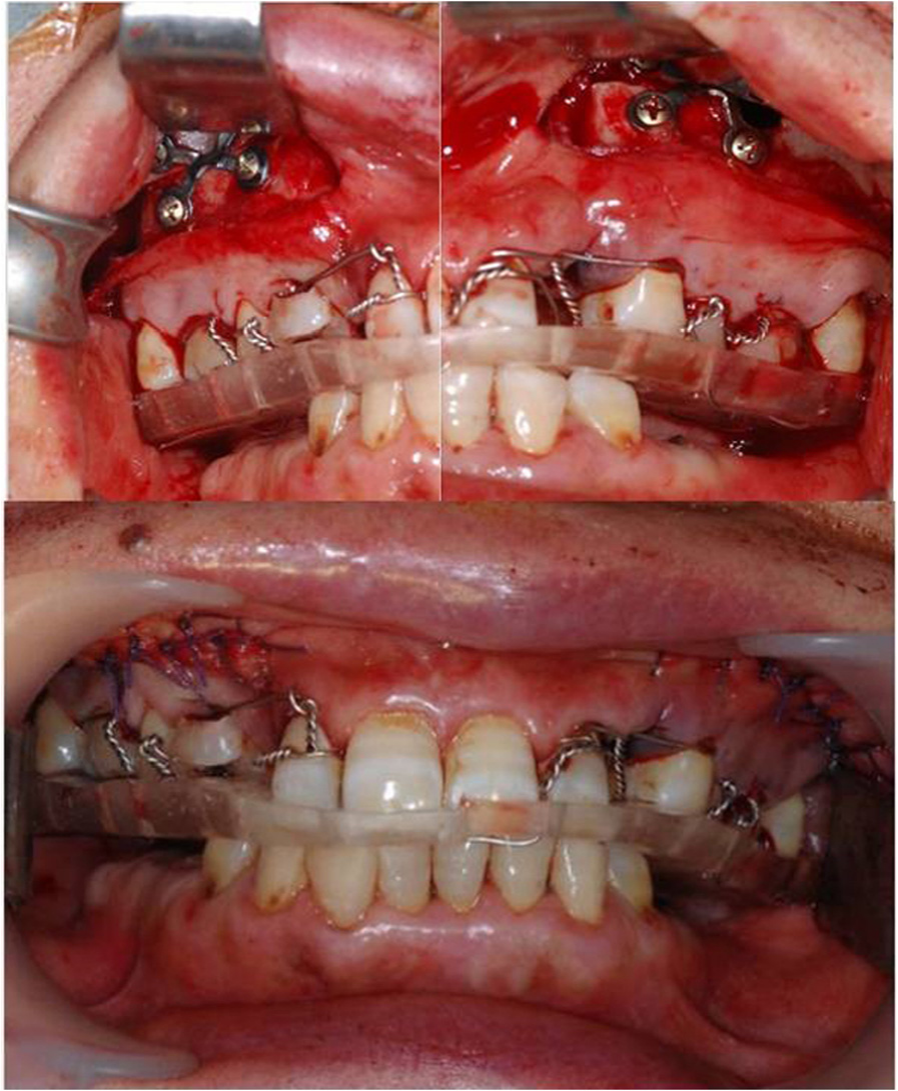
Intraoperative photo of case 1 showing a wafer in place

After palatal osteotomy, the maxillary posterior segment was separated, and excessive bone fragments were removed. The segmented bone fragments were shifted using the wafer, which was made before operation, and then they were fixed using the mini plate and screws. And the operation was finished following intra-oral suture and intermaxillary fixation (Fig. 2).

**Fig. 3:**
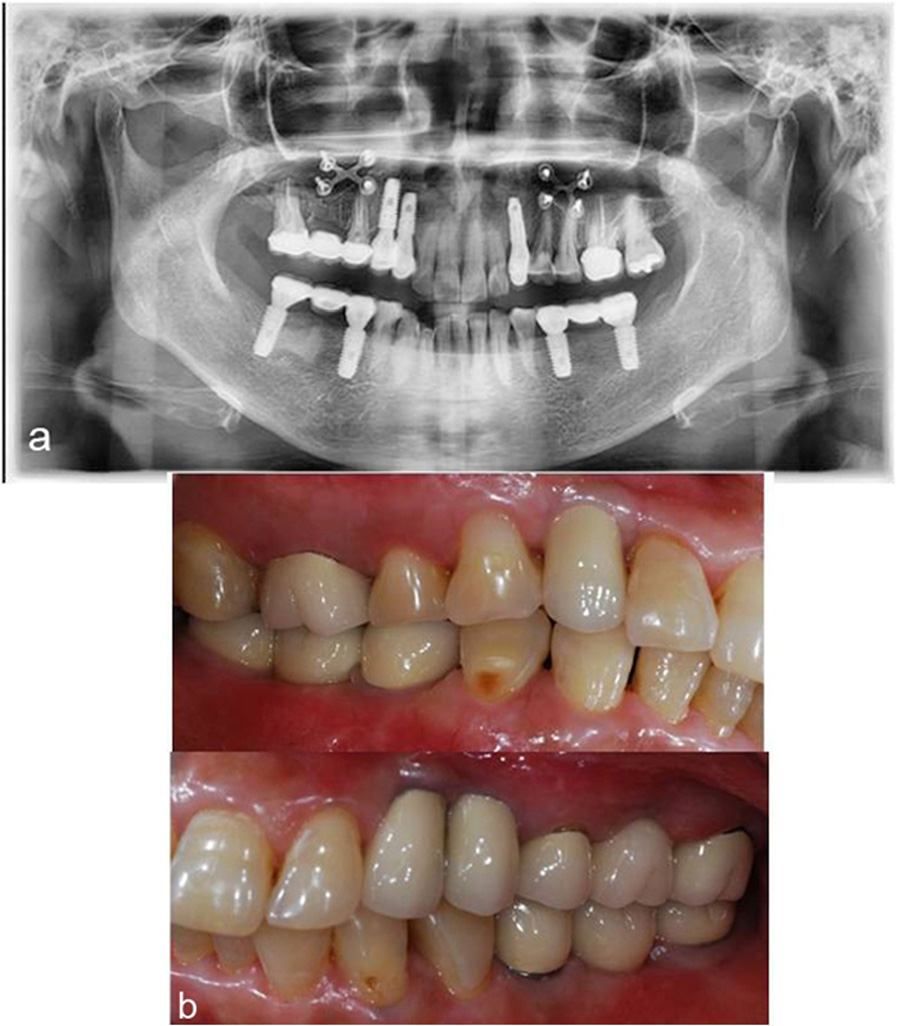
Postoperative panoramic view (**a**) and intra-oral photo of case 1 showing increased interarch space by implant-supported FPD getting a mean 6-mm clinical crown length of the mandibular molar (**b**)

At 2 weeks, stitch-out and removal of the intermaxillary fixation were conducted, and at 4 weeks, the wafer placed on the maxillary teeth was shifted. Then, implant placement and prosthetic treatment were done (Fig. 3). One, 2, and 6 months after the surgery, we confirmed stable occlusion and no complications such as necrosis of the osteotomy site.

### Case 2

**Fig. 4:**
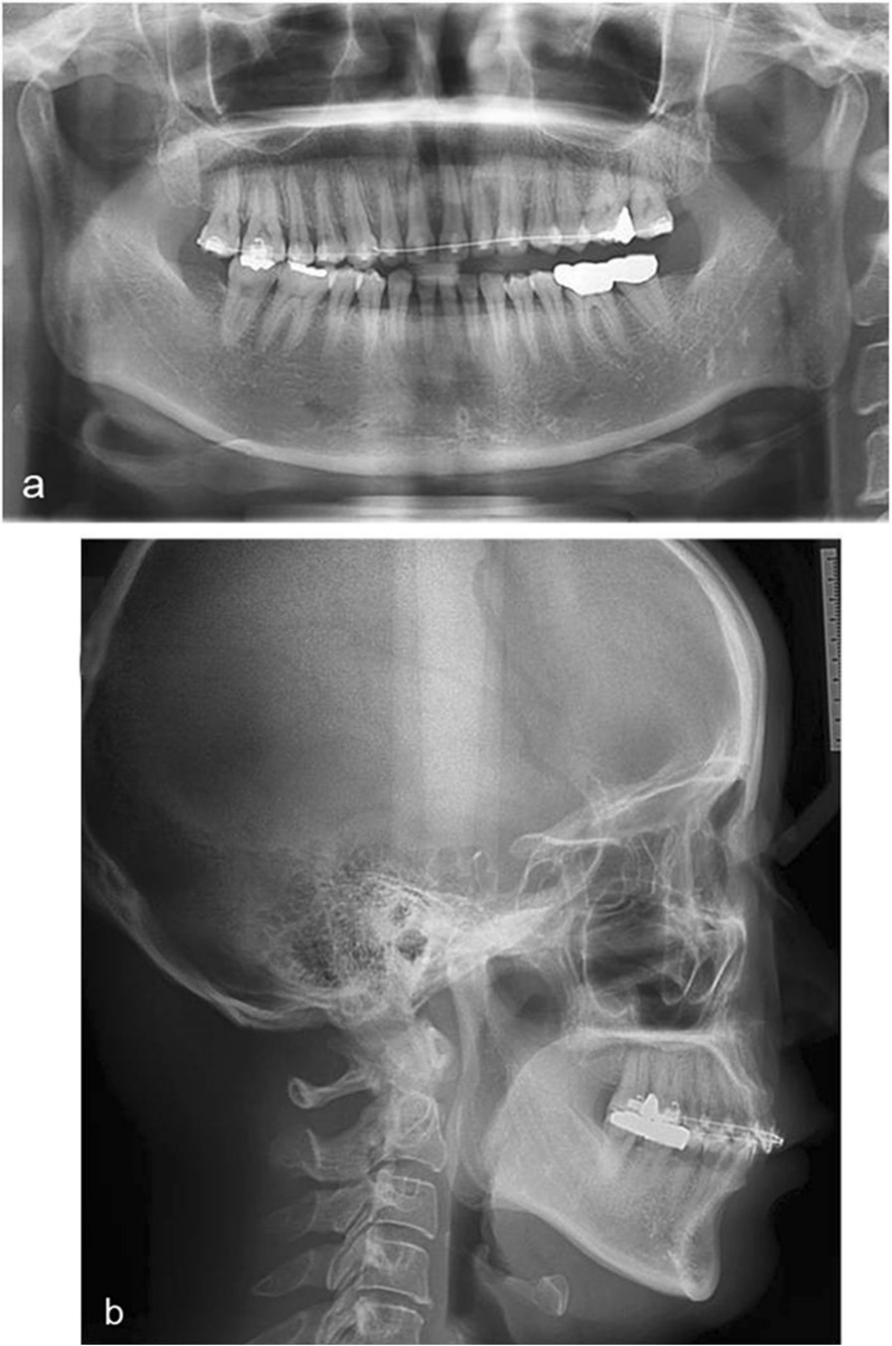
Preperative panoramic view showing decreased vertical dimension (**a**) and cephalometirc X-ray of case 2 showing retruded maxilla and mandible (**b**)

**Fig. 5:**
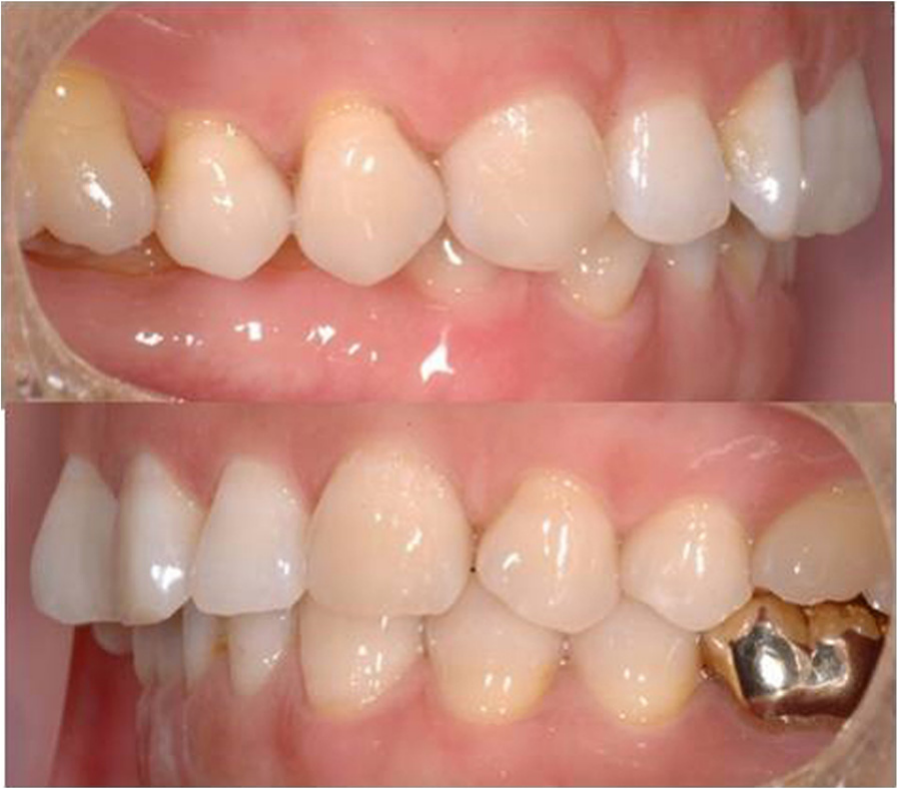
Preoperative intra-oral photo of case 2 showing collapsed right posterior maxillary area

**Fig. 6:**
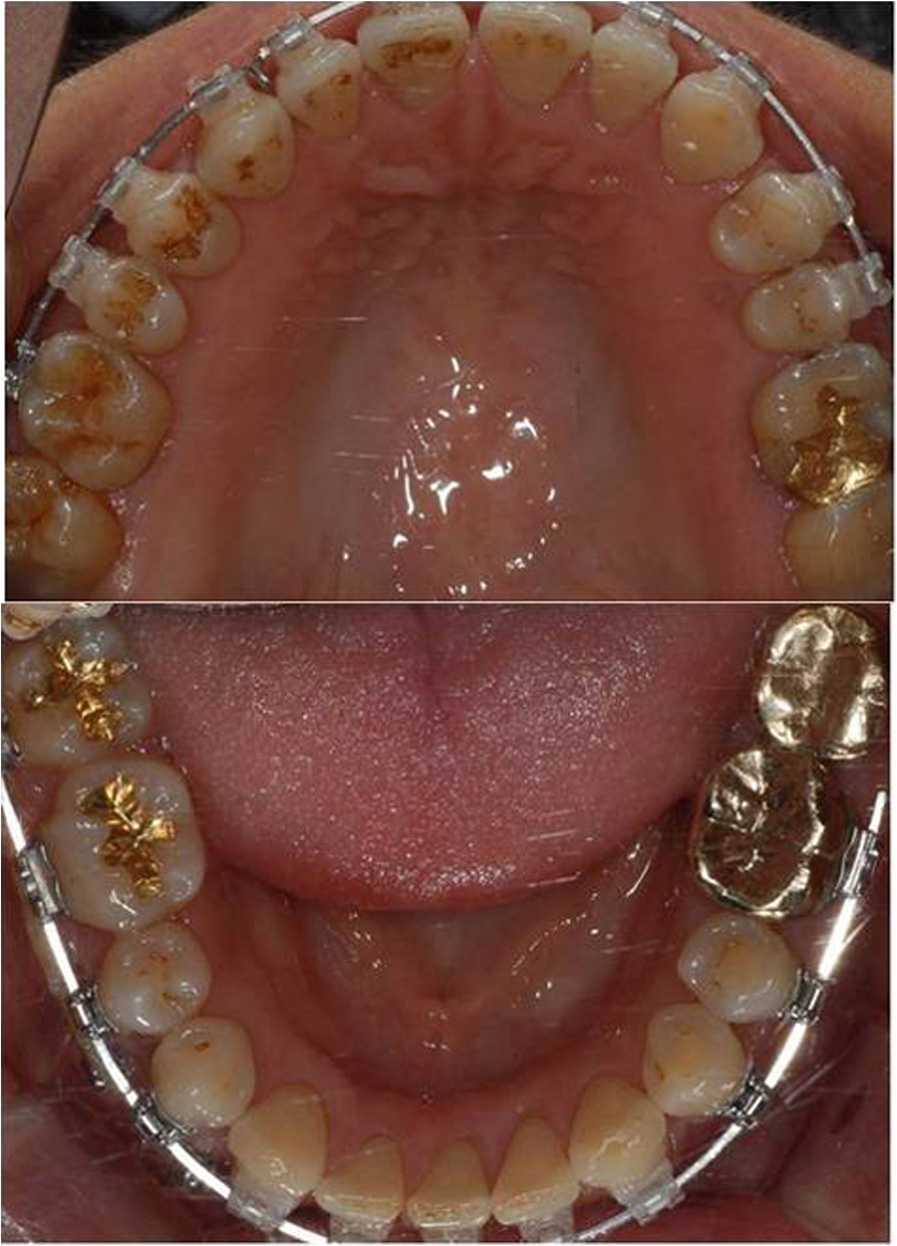
Preoperative intra-oral photo of case 2 showing discrepancy of maxillary right intermolar width (maxillary inter molar width 46 mm, mandibular intermolar width 40 mm)

A 43-year-old female patient visited the hospital for treatment of the erupted maxillary right molar and the molar width discrepancy and protruded upper lip. She was able to bite just in the left due to excessive eruption (downward canting, 3 mm) of the maxillary right molar and scissor bite in clinical and radiological examinations (Figs. 4 and 5). Over eruption and scissor bite of right maxillary molar teeth, maxillary and mandibular hypo-growth (SNA 75.68, SNB 71.41), maxillary arch length discrepancy (maxillary inter molar width 46 mm, mandibular intermolar width 40 mm) (Fig. 6), acute nasolabial angle (90°), and protruded upper lip (upper lip E-line 1.95) were found, and for surgical treatment, Le Fort I osteotomy and PMSO were performed. According to the analysis and surgical planning, total setback 5.5 mm, posterior impaction 3.0 mm, and medial shift of maxillary right molar 5.0 mm was planned.

Le Fort I osteotomy was conducted in accordance with local analgesia under general anesthesia.

**Fig. 7:**
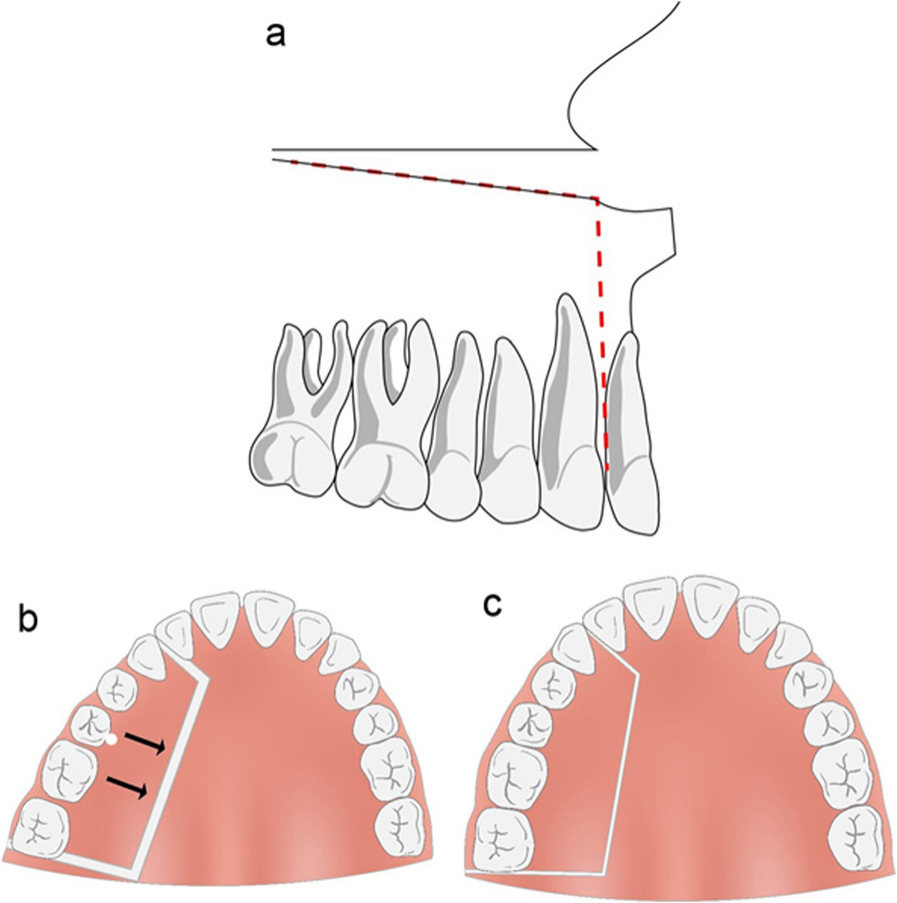
Schematic drawing of Le Fort I osteotomy and posterior segmental osteotomy. **a** Le Fort I down fracture and vertical osteotomy line of posterior segmental osteotomy. **b** Preoperative state of palatal side showing osteotomy line. **c** Postoperative state of palatal side that corrected intermolar width

After maxillary down fracture was performed, maxillary setback was secured using the intermediate wafer. And then, vertical osteotomy and palatal osteotomy were conducted between the maxillary right lateral incisor and maxillary right canine, and osteotomy of the maxillary right molar was finished (Fig. 7). Medial shift of the segmental bone fragment was identified using the final wafer, and then it was fixed using the plate and screws, and intra-oral suture and intermaxillary fixation were carried out (SNA 67.66, SNB 66.41).

**Fig. 8:**
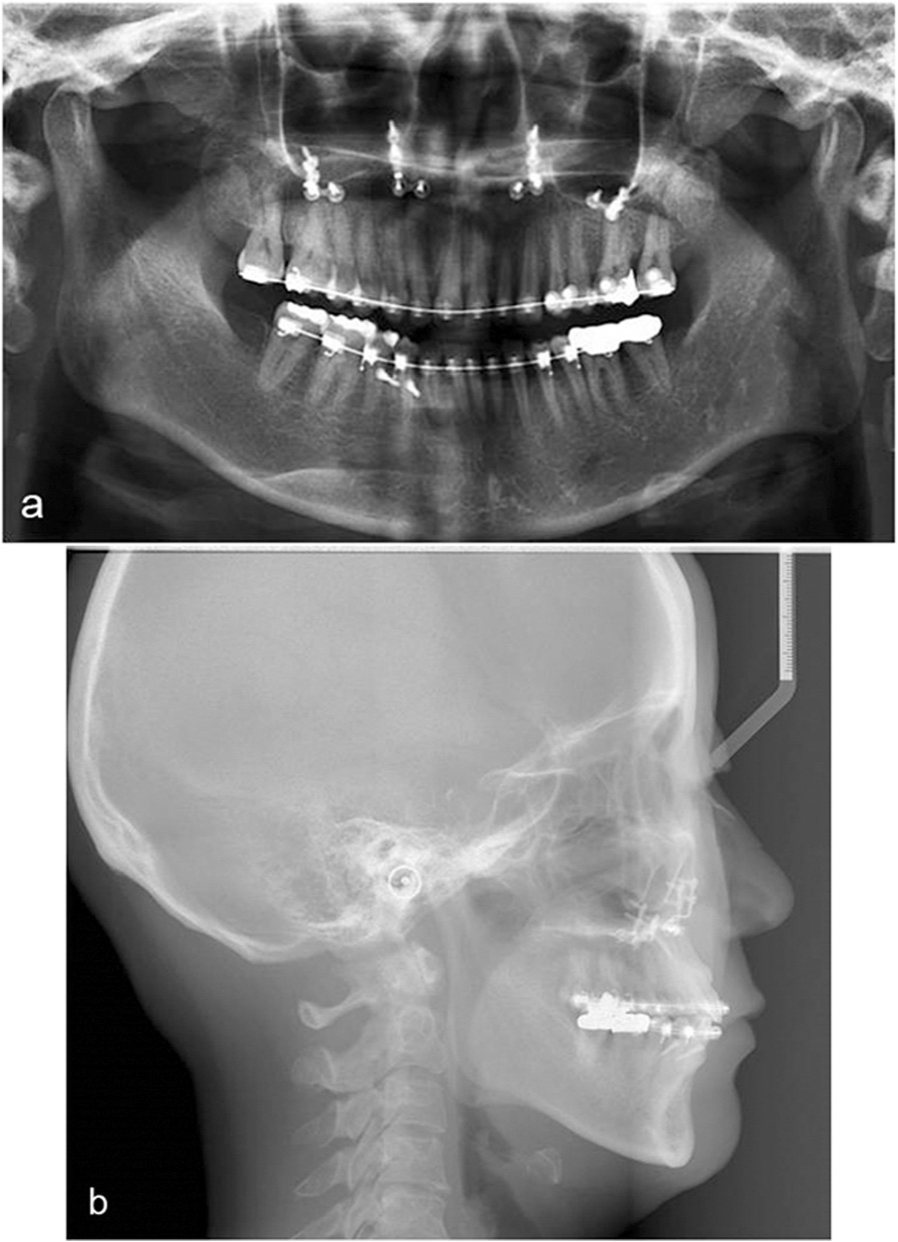
Postoperative panoramic view showing increased vertical dimension (**a**) and cephalometirc X-ray of case 2 showing increased interarch space of right posterior maxillary area (**b**)

**Fig. 9:**
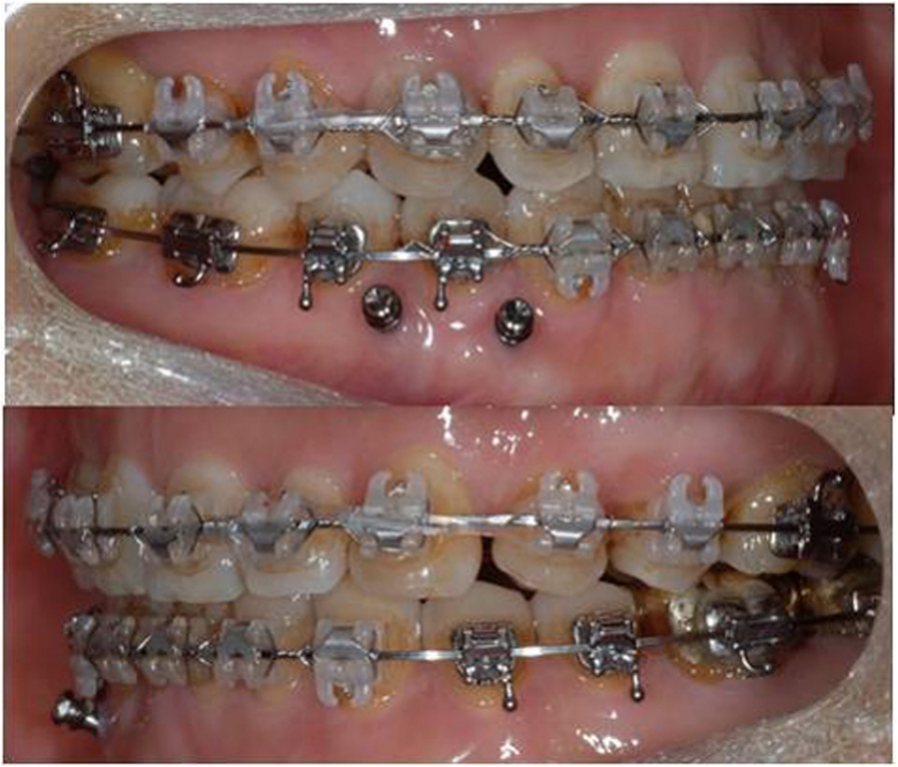
Postoperative intra-oral photo of case 2 showing increased vertical dimension

**Fig. 10:**
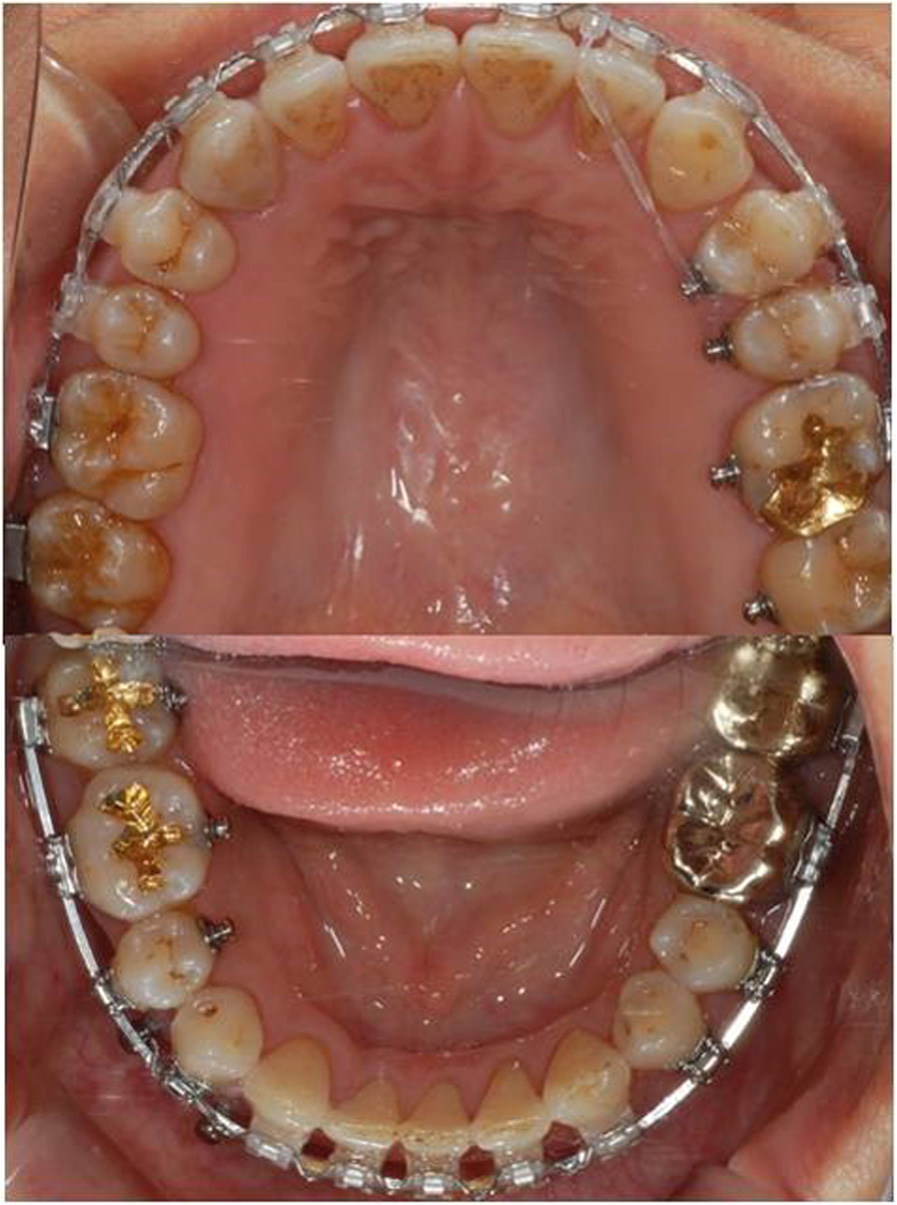
Postoperative intra-oral photo of case 2 showing corrected inter molar width of maxilla (maxillary inter molar width 41 mm, mandibular inter molar width 40 mm)

At 2 weeks, stitch-out and intermaxillary fixation were removed, and at 4 weeks, the wafer was removed. She continued postoperative orthodontic treatment and she had stable occlusion (Figs. 8 and 9). Maxillary arch length discrepancy (Fig. 10) was also effectively decreased (maxillary inter molar width 41 mm, mandibular inter molar width 40 mm), enabling the patient to chew with bilateral molars and satisfying the lateral profile of the upper lip (upper lip E-line −1.82).

### Discussion

Segmental osteotomy is a surgical procedure which moves alveolar bone fragments of the teeth to improve skeletal malformation and malocclusion and is divided into anterior and posterior molar segmental osteotomy according to the location of operation [6, 7].

Of these, posterior maxillary molar segmental osteotomy was developed by Schuchardt as a two-stage operation for the treatment of anterior open bite in 1954, and then it was transformed into a one-stage operation by Kufner in 1960. Since post-PMSO physiological cure was reported by Bell in 1971, the one-stage operation has been broadly used [8, 9].

If intermaxillary space is decreased by the erupted maxillary molar for a variety of causes, various treatment methods can be attempted so that the patient can bite normally. However, if the degree of eruption is serious, there may be limitations in establishing an ideal treatment plan with prosthetic treatment alone.

Forced orthodontic intrusion using extruded molar is also a good treatment option and has been shown satisfactory results [10]. If one proceeds with orthodontic treatment alone to solve the decreased vertical dimension or arch length discrepancy, it may cause various problems. Patient compliance may be difficult to achieve given the significant length of treatment. Root resorption routinely occurs when forced intrusion is proceeded. The extrusion of anchorage teeth is the main complication of the conventional orthodontic method such as intrusion arch technique [11]. And there are still limitations in the amount of intrusion even when using contemporary method such as micro-implant. The variation of the maxillary molar intrusion ranged from −3.68 to 8.67 mm accompanying buccal tilting of the compromised molar teeth [12].

PMSO can be used as a surgical procedure for securing decreased intermaxillary space, treatment of horizontally excessive growth of the maxilla, maxillary and mandibular arch width discrepancy, molar open bite, and deep bite [1]. And it can rather be a conservative approach in terms of saving the severely extruded molar teeth.

In this case report, the patients lacked intermaxillary space due to the seriously erupted maxillary molar. So, proper occlusal rehabilitation only by prosthetic treatment was difficult. Also, as cross bite of the molars exists, molar relationship can be improved by orthodontic treatment, but limited shift inevitably occurs because tooth shift is only possible within the alveolar housing during orthodontic treatment. After attempts to secure intermaxillary space and balance interarch molar width were made through maxillary molar segmental osteotomy in such situations, their biting ability could be successfully restored.

To the best of the authors’ knowledge, the posterior maxillary osteotomy is not performed only for posterior molar width improvements. It is usually used as a preemptive treatment to improve the prosthetic problems that appear after tooth extraction is accompanied. However, when there were difficulties before making implant crown after implant surgery due to unbalanced molar width following excessively tilted implant to the buccal side, satisfactory outcome was obtained by correcting the implant location through maxillary molar segmental osteotomy in some cases [3]. Therefore, this case report is meaningful in that maxillary molar segmental osteotomy was used to improve decreased arch space and improper molar width following tooth extraction.

Implant placement of the mandibular molar can be done at the same time with maxillary molar segmental osteotomy, or can be done after the operation, but the proper timing has not been established clearly yet [4, 5, 13]. If operation is conducted simultaneously, a treatment period can be shortened and frequency of operation can be decreased. However, in this case report, the patients wanted a less invasive treatment method, so PMSO and implant placement of the mandibular molar could not be simultaneously performed.

As complications of maxillary molar segmental osteotomy, postoperative infection of the surgical site, hemorrhage, vitality loss of the adjacent tooth, and necrosis of bone fragments may occur. Of these, in particular, damaged dental root adjacent to the surgical site may be fully prevented by the operator, so care must be taken. Rarely, cases of inflammatory dental root resorption have been reported after a long time following maxillary molar segmental osteotomy [14]. On the other hand, according to the histological study by Lownie et al., pulpal tissues of the adjacent tooth following segmental osteotomy were spontaneously cured; therefore, endodontic treatment of the dental root adjacent to the surgical site is not essential and may be delayed until a clinical symptom appears [15–17]. Accordingly, postoperative pulp vitality test should be accompanied. In this case report, neither pulpal necrosis on the adjacent tooth of the region where segmental osteotomy was conducted nor the accompanying symptoms occurred.

The success or failure of the segmental osteotomy is dependent on the ongoing blood supply to the segmented bone. An unusual complication of the PMSO is the open bite caused by insufficient osteotomy. It is important to keep the integrity of the palatal mucosa of the mobilized segment unless it leads to the avascular necrosis of the segmented bone. During the hospitalization and follow-up periods, we did not find any irregularity of the palatal mucosa and avascular necrosis of the operated site, and the occlusion was stabilized without the open bite.

## Conclusions

This clinical case report describes surgical intervention of maxillary molar segmental osteotomy accompanied with orthodontic treatment and implant-supported prosthetic treatment to restore decreased vertical dimension of the molar and improve unbalanced molar width. It can be a successful treatment method, and further studies are required on the application of PMSO in order to improve molar width discrepancy.
